# An Alternative Non-Invasive Screening Model for Liver Fibrosis among US Adults at Risk of MASLD

**DOI:** 10.3390/diseases12070150

**Published:** 2024-07-11

**Authors:** Hongbing Sun

**Affiliations:** Nutrition, Biostatistics and Health Study, Department of Earth and Chemical Sciences, Rider University, 2083 Lawrenceville Road, Lawrenceville, NJ 08648, USA; hsun@rider.edu; Tel.: +1-609-896-5185; Fax: +1-609-895-5782

**Keywords:** LSS model, screening for liver fibrosis, liver stiffness score, advanced liver fibrosis

## Abstract

**Background and Aims:** Screening for liver fibrosis presents a clinical challenge. This study aimed to explore a useful alternative method for assessing fibrosis risk among US adults at risk of metabolic dysfunction-associated steatotic liver disease (MASLD). **Methods:** A liver stiffness score (LSS) model was proposed and tested using data from 3976 participants at possible risk of MASLD, obtained from the US National Health and Nutrition Examination Survey (NHANES). **Results:** The LSS model was developed using liver stiffness measurements, blood biochemistry, and body measurement data from 2414 NHANES participants at risk of MASLD, sampled between 2017 and 2020: LSS = exp(0.007035 × bodyweight_kg_ − 0.1061 × race_black1,0_ + 0.183221 × diabetes_1,0_ + 0.008539 × AST_IU/L_ − 0.0018 × plateletcount_1000cell/UL_ − 0.21011 × albumin_g/dL_ + 2.259087). The probability (P) of having fibrosis F3 + F4 is calculated as follows: P = 0.0091 × LSS^2^ − 0.0791 × LSS + 0.1933. The developed LSS model was tested on 1562 at-risk participants from the 2017–2018 cycle. The results showed that the LSS model achieved AUROC values of 0.79 and 0.78 for diagnosing cirrhosis (F4) and advanced fibrosis (F3 + F4) in the US population, respectively. It outperformed existing models such as NFS, FIB-4, SAFE, and FIB-3. For screening F3 + F4 fibrosis, the LSS model’s top decile outperformed the NFS and FIB-4 models by 37.7% and 42.6%, respectively. Additionally, it showed superior performance compared to the waist circumference classification method by 29.5%. **Conclusions:** derived from an ethnically diverse population dataset, the LSS screening model, along with its probability equation, may offer clinicians a valuable alternative method for assessing the risk of liver fibrosis in the at-risk adult population.

## 1. Introduction

Screening for liver disease before the onset of complications presents a significant challenge in clinical practice [1]. Patients often show no symptoms or specific biological abnormalities until complications arise, leading to delayed diagnosis and disease progression. This delay can ultimately result in liver decompensation or palliative hepatocellular carcinoma, with a poor medium-term prognosis [2,3]. While liver biopsy remains the gold standard for assessing liver fibrosis, its invasive nature, cost, and potential for complications render it impractical as a screening tool for the general population [4,5]. Similarly, non-invasive elastography, though less invasive, may not be ideal for population-wide screening due to its high acquisition and maintenance costs and limited access in primary care settings [6]. Conversely, models that utilize routine clinical data and blood test results hold promise for widespread and repeated use at the population level, as they can be readily employed by any physician [7,8,9].

Fibrosis-4 (FIB-4) [10] and Non-Alcoholic Fatty Liver Disease (NAFLD) Fibrosis Score (NFS) [11] are two of the most commonly used models for screening advanced fibrosis through simple blood tests among the population at risk of NAFLD, currently metabolic dysfunction-associated steatotic liver disease (MASLD)-related fibrosis [12]. Both models utilize the liver enzymes alanine transaminase (ALT) and aspartate transaminase (AST), platelet count, and age in their calculations, with NFS additionally incorporating hyperglycemia, body-mass index, and albumin. While both FIB-4 and NFS have been validated for identifying advanced fibrosis in multiple studies [7,8,9], recent research has also raised concerns regarding their lower accuracy as screening tools for liver fibrosis in the at-risk population for MASLD (simplified as at-risk population from here on) [12,13,14]. These recent findings suggest a potential need for an alternative approach to enhance the performance of these modeling methods in the at-risk population.

Therefore, the objective of this study was to develop a useful alternative liver stiffness score (LSS) model using routine laboratory and clinically available variables for the at-risk population, similar to the goals of the FIB-4 and NFS models. This model will be developed using the relationship between FibroScan liver stiffness measurements (LSMs) and body measurements and blood biochemistry data obtained from participants of the US National Health and Nutrition Examination Survey (NHANES) during the 2017–2020 sample cycle. Subsequently, it will be tested using LSM data from participants of the 2017–2018 sample cycle.

To the best of our knowledge, the NHANES dataset [15] stands out as the only dataset with a sophisticated sampling design that represents the ethnically diverse US population. The NHANES LSM dataset may be uniquely suited for deriving a screening model for liver fibrosis in the population.

## 2. Data and Method

A total of 3976 NHANES participants were identified as at risk for MASLD-related fibrosis, comprising 2414 from the 2017–2020 sample cycle and 1562 from the 2017–2018 sample cycle, out of 13,764 adult NHANES participants who had FibroScan LSM data (5232 from the 2017–2018 cycle and 8532 from the 2017–2020 cycle) [15]. The participants of the 2017–2020 and 2017–2018 sample cycles are different, despite overlapping years. The 2019–2020 pre-pandemic sample was combined with data from the NHANES 2017–2018 cycle to form a nationally representative sample of 2017–2020 sample data due to incomplete sample collection in the 2019–2020 sample cycle.

The selection of the 3976 participants at risk of MASLD followed the American Association for the Study of Liver Diseases (AASLD) Practice Guidance for screening advanced fibrosis [16,17]. A more detailed step-by-step selection process using the current dataset was also described in a previous study [12]. To be included, a participant needed to have prediabetes or type 2 diabetes, elevated ALT levels > 30 U/L or AST levels > 30 U/L, and at least two of the following metabolic factors: obesity, as determined by waist circumference criteria (Asian male ≥ 90 cm, males of all other ethnicities ≥94 cm, all females ≥ 80 cm); serum triglyceride levels ≥ 150 mg/dL; serum high-density lipoprotein (HDL) cholesterol levels below specified thresholds (<40 mg/dL in men, <50 mg/dL in women); hypertension, with specified blood pressure criteria (systolic blood pressure ≥ 130 mm Hg or diastolic blood pressure ≥ 85 mm Hg or higher); fasting plasma glucose levels ≥ 100 mg/dL.

Participants were excluded if they had acute or chronic viral hepatitis B or C, as per American Gastroenterological Association (AGA)/American Association for the Study of Liver Diseases (AASLD) guidance [16,17], and if their ALT and AST values exceeded 500 IU/L (excluding possible paracetamol overdose). Additionally, individuals with missing alcohol consumption data were excluded, along with men reporting more than 3 drinks and women reporting more than 2 drinks per day on average over the past 12 months before their interview [12]. Participants with unreliable LSM data, defined as an interquartile range of liver stiffness to the median (IQR/M) > 30% and a median LSM ≥ 7.1 kPa, were also excluded [18]. Those diagnosed with congestive heart failure [19], as well as those with missing body weight, waist circumference, or liver AST, blood platelet count, and albumin data, were excluded as well.

The diabetes status of participants was determined using the following criteria, following prior practices [20,21,22]: self-report, random glucose test results ≥ 200 mg/dL, fasting glucose levels ≥ 126 mg/dL, or HbA1c levels ≥ 6.5% or the use of glucose-lowering medications such as sulfonylureas, insulin, or incretin mimetics. The use of metformin alone was not considered diagnostic of diabetes due to its potential use in the prediabetic population. However, users of metformin were included as prediabetic, as well as those with A1C levels ≥ 5.7% and <6.5%, if they were not identified as diabetic per the above definition. The separation of diabetes and prediabetes here was because diabetes was used as one of the variables in the LSS model proposed in this study. The 3976 at-risk participants identified in our study versus the 3741 identified by Chang et al. [12] from this same group might arise from the varied criteria for identifying diabetes in our approach.

## 3. Outcome and Statistical Analyses

The outcome of this study is the creation of a non-linear multivariate regression model for estimating LSS using routine laboratory data from the cohort of 2414 at-risk participants from the 2017–2020 sample cycle, along with the formulation of a method to estimate the probability risk of fibrosis among the at-risk patients. The selection of parameters and estimation of coefficients involved multiple stepwise trial-and-error analyses. Coefficients for the LSS regression model were determined using the measured LSM values of the 2414 participants as the dependent variable. Independent variables included AST, platelet count, albumin, body weight, diabetes status, and race. The probability formulas for participants with fibrosis stages (or scores) of F2–F4 (or ≥F2), F3 + F4, and F4 were derived from the relationship between median LSS values and the likelihood of fibrosis within each of the 20 sequential LSS quantiles. Twenty quantiles were used because this number of quantiles maintains sensitivity and reduces fluctuations of the plotted trend line compared to when the quantile number is either too small or too large. Fibrosis classification was based on LSM ranges: 2–7 kPa as F0–F1, 7–7.5 kPa as transitional (Ft), 7.5–10 kPa as F2, 10.1–14 kPa as F3, and above 14 kPa as F4 [23,24].

The performances of the models were evaluated using data from 1562 at-risk participants from the 2017–2018 sample cycle. Assessment criteria included comparing the area under the receiver operating characteristic curves (AUROCs) of the LSS, NFS, FIB-4, Steatosis-associated fibrosis estimator (SAFE) [25], and Fibrosis-3 Index (FIB-3) [26] models for diagnosing fibrosis. In addition, the predicted probabilities of having fibrosis within subdivided groups were compared to actual proportions based on LSM, along with the percentage of participants with fibrosis placed in their corresponding deciles.

Multivariate non-linear regressions and ROC analyses were conducted using the Non-linear Regression model and ROC Regression model in Stata (version 18.0, StataCorp LLC, College Station, TX, USA). A *p*-value ≤ 0.05 was considered as the threshold for statistical significance.

## 4. Results

In the group of 2414 at-risk participants from the 2017–2020 sample cycle, 19.6% (Table 1) showed possible fibrosis (LSM > 7.5 kPa).

### 4.1. Introduction of the Liver Stiffness Score (LSS) Model

A non-linear multivariate regression model for predicting LSS, utilizing measured LSM values from 2414 at-risk participants from the 2017–2020 sample cycle as the dependent variable, and AST, body weight, platelet count, diabetes status, albumin, and race as independent variables, is presented as follows:

LSS = exp(0.007035 × bodyweight_kg_ − 0.1061 × race_black1,0_ + 0.183221 × diabetes_1,0_ + 0.008539 × AST_IU/L_ − 0.0018 × plateletcount_1000cell/UL_ − 0.21011 × albumin_g/dL_ + 2.259087) (1)

where:

bodyweight_kg_ represents the weight of the body in kilograms;

race_black1,0_ indicates if the race is black, race = 1; if not, race = 0;

diabetes_1,0_ indicates if there is a diabetes, diabetes = 1; if not, diabetes = 0;

liver AST_IU/L_ has a unit of IU/L;

plateletcount _1000cell/UL_ indicates platelet count has a unit of 1000 cells/UL;

albumin has a unit of g/dL;

exp denotes the natural exponential function.

**Figure 1:**
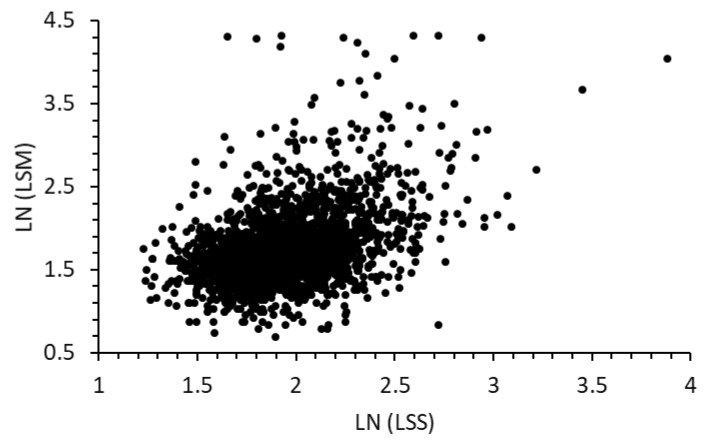
Scatter plot of natural log values of predicted LSS versus measured LSM data (2017–2020 data).

The regression result, known as LSS, was not assigned a unit here to distinguish it from FibroScan LSM (kPa). Though the values of the calculated LSS correlated significantly with the measured LSM data, their differences are significant in the log–log plot (Figure 1). For diagnosing fibrosis F4, based on the LSM of the 2414 at-risk participants, the AUROC is 0.79 (95% CI: 0.73–0.84). Using an optimal cutoff [27] of 7.3, the sensitivity is 77.8%, and the 1-specificity is 37.8%.

### 4.2. Fibrosis Probabilities with the New LSS Model

**Figure 2:**
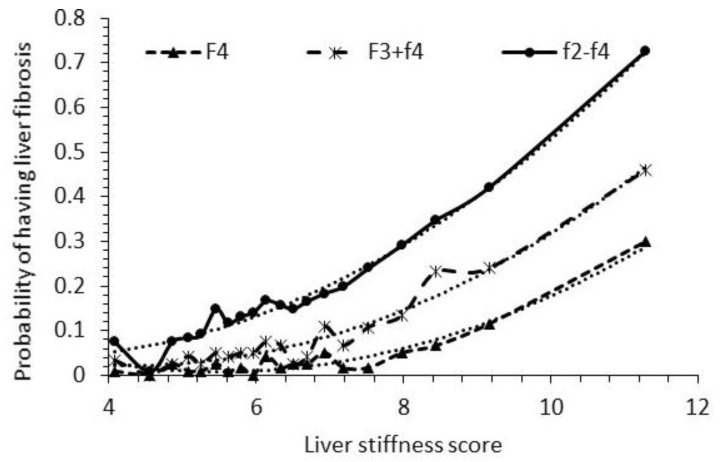
Probabilities of participants having F2–F4, F3 + F4, and F4 versus the median liver stiffness score (LSS) of 2017–2020 participants. The figure includes fitted trend lines (dotted lines).

The LSS values of 2414 participants were divided into 20 sequential quantiles from the lowest to the highest. The median LSS values of each of the 20 quantiles versus the probabilities (the percentage) of F2–F4, F3 + F4, and F4 falling into each of these 20 quantiles are plotted in Figure 2. Additionally, their trend formulas, which denote the probability of having fibrosis corresponding to an LSS value, are provided here.

Probability of having F2–F4 = 0.0098 × LSS^2^ − 0.0579 × LSS + 0.127 (2)

Probability of having F3 + F4 = 0.0091 × LSS^2^ − 0.0791 × LSS + 0.1933 (3)

Probability of having F4 = 0.0084 × LSS^2^ − 0.0925 × LSS + 0.2631 (4)

The R^2^ values of the fittings in Figure 2 are 0.983, 0.958, and 0.951 for Equations (2), (3) and (4), respectively. The probabilities of Equations (2)–(4) would be approximately 1 when LSS > 12.9, >14.8, and >16.5 for F2–F4, F3 + F4, and F4, respectively. One can estimate the probability of having F2–F4, F3 + F4, and F4 based on Equations (2) to (4). For example, if a patient’s LSS value is 10, the probability of the patient having fibrosis F2–F4 would be 67.6%, fibrosis F3 + F4 43.4%, and fibrosis F4 alone 26.2%, using the above formulas. The probability of having fibrosis increases as the LSS value increases.

### 4.3. Assessing Model Validity: Testing with Data from 2017–2018 Sample Cycle of 1562 at-Risk Participants

**Figure 3:**
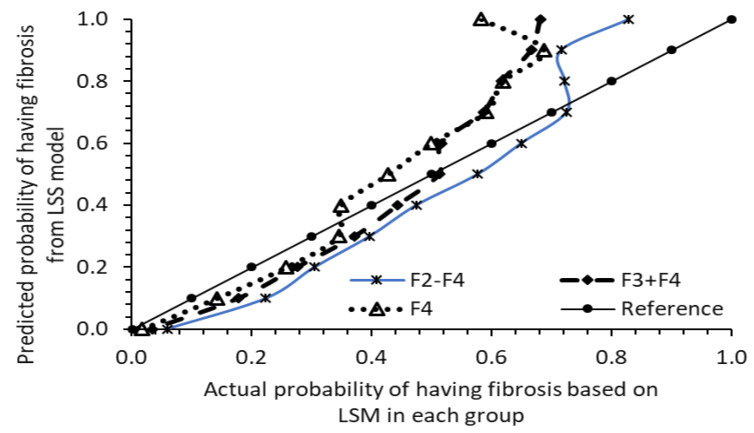
Predicted probability of having fibrosis from LSS model versus actual probability of having fibrosis (F2–F4), (F3 + F4), and (F4) based on LSM in 1562 participants in each probability group (2017–2018 sample).

To assess the model’s validity, LSS values and probabilities of having fibrosis F2–F4, F3 + F4, and F4 were calculated for 1562 at-risk participants using Equation (1) and subsequently (2)–(4). These participants were then categorized into 11 groups based on their probability of having fibrosis: >0.999, >0.9, >0.8… >0.1, and <0.1. The ratios of participants with fibrosis F2–F4, F3 + F4, and F4 in each probability group to the total number of participants in that group were plotted against the calculated probability from the LSS model’s formula (Figure 3). It is evident that the model performed well in probability prediction up to 0.7 for F2–F4, 0.5 for F3 + F4, and 0.35 for F4. Beyond these probability thresholds, the predicted probability exceeded the actual risk probability of the group based on the ratio of counts from LSM. These validity ranges align with the probability derivation shown in Figure 2, where no data points extend beyond the probability levels of 0.7 for F2–F4, 0.5 for F3 + F4, and 0.35 for F4.

## 5. Discussion

Both AGA and AASLD recommend screening for liver fibrosis in at-risk patients [16,17]. Yet among the at-risk NHANES participants with possible fibrosis (LSM > 7.5 kPa), 84.4% (95% CI 80.8% to 87.4%) had never received a diagnosis of a liver condition from a physician. Similarly, 78.7% (95% CI 72.8% to 83.5%) and 73.7% (95% CI 64.2% to 81.5%) of those with possible F3 + F4 and F4 fibrosis, respectively, had never been informed about their liver condition by a physician. These findings underscore the importance of screening for fibrosis in the at-risk US population.

### 5.1. FIB-4 and NFS Models: Screening for Fibrosis and Concerns

FIB-4 and NFS models are the two most widely used non-invasive screening tests for predicting advanced fibrosis in MASLD (formerly NAFLD) patients, validated through numerous studies comparing them with liver biopsy data [7,8]. The 2021 AGA Clinical Care Pathway and 2023 AASLD Practice Guidance suggest that patients identified with indeterminate risk through FIB-4 screening be referred for FibroScan evaluation [16,17]. The NFS model has also received endorsement from both the European Association for the Study of the Liver and the AASLD for screening advanced fibrosis in patients with MASLD [28,29].

Despite these recognitions, recent concerns about the screening efficiency of both FIB-4 and NFS models in fibrosis screening are significant. Chang et al. [12] found that 79.1% of subjects classified as high risk by FIB-4 were not high risk based on LSM, with 62.8% being low risk. Graupera et al. [13] reported that nearly one-third of elevated FIB-4/NFS results were false positives. Mauro Viganò et al. [14] reported FIB-4’s suboptimal performance in referring patients to liver centers, with about one-fifth being falsely negative. The current study also revealed lower accuracy of both models in screening for F3 + F4 compared to waist circumference alone, supporting Graupera et al.’s findings [13]. Therefore, it seems an alternative method might need to be explored.

### 5.2. Comparison of LSS Model with FIB-4, NFS, SAFE, and Waist Circumference for Fibrosis Screening

**Figure 4:**
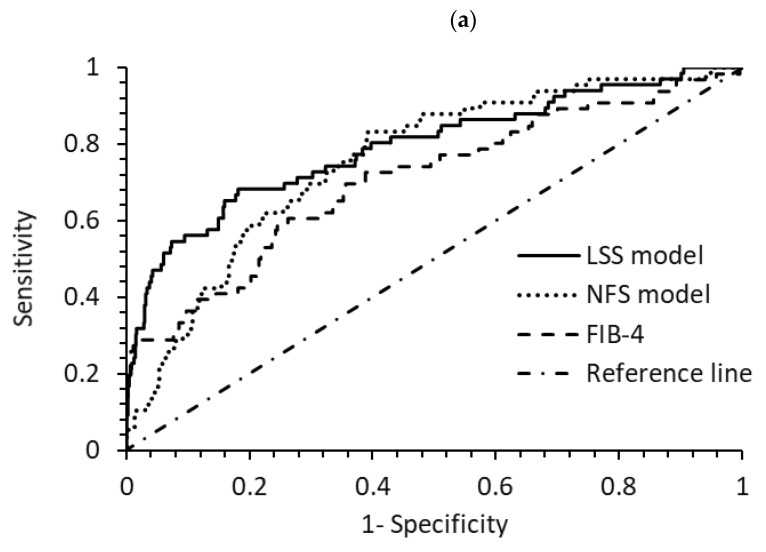

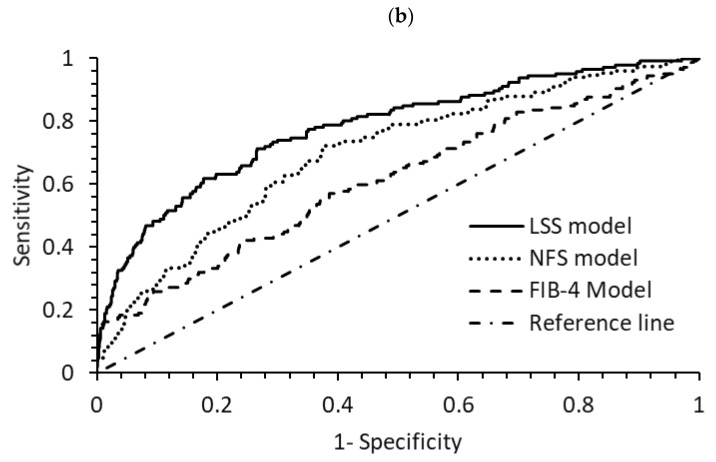
Comparisons of receiver operating characteristic curves for diagnosing (**a**) cirrhosis F4 and (**b**) advanced fibrosis F3 + F4 among three models. N = 1562, 2017–2018 sample.

The LSS model exhibited an improved AUROC compared to the FIB-4, NFS, SAFE [23], and FIB-3 [24] models for diagnosing cirrhosis (F4) in 1562 at-risk participants based on LSM data (Table 2). These differences were not statistically significant (Figure 4a). However, for diagnosing advanced fibrosis (F3 + F4), the LSS model significantly outperformed the NFS, FIB-4, and FIB-3 models. For diagnosing significant and advanced fibrosis (F2–F4), the LSS model significantly outperformed all four models: NFS, FIB-4, SAFE [23], and FIB-3 [24] (Table 2). The SAFE model, developed for the general population, and FIB-3, tailored for the NAFLD group from the Japanese population, are more recent models for screening fibrosis. The improved performance of the LSS model suggests that it is likely more efficient in screening for all stages of fibrosis among the at-risk population compared to the aforementioned models.

**Figure 5:**
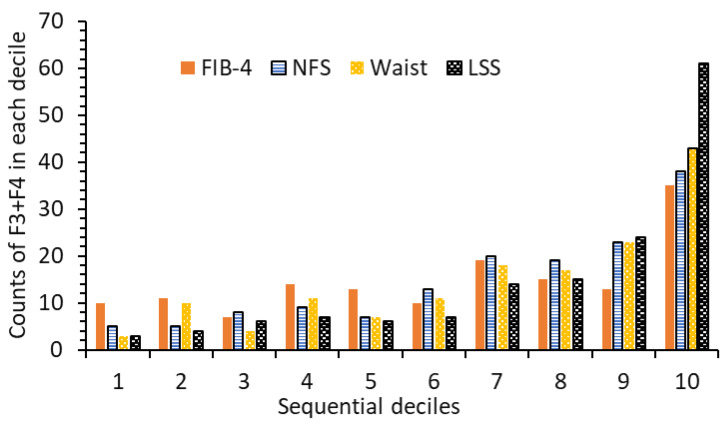
Counts of participants with F3 + F4 falling in sequential deciles of LSS, FIB-4, NFS models, and waist circumferences (2017–2018 sample).

A recent study also found that waist circumference alone performed better than both the FIB-4 and NFS models in screening for fibrosis among risk groups [13]. To further compare the new LSS model and the two established models, NFS and FIB-4, and to evaluate the claim of waist circumference outperforming for screening advanced fibrosis (F3 + F4), 1562 participants were divided into corresponding sequential deciles based on their values from each model (Figure 5). The number of participants with fibrosis F2, F3, and F4 within each decile of the corresponding models was counted. In the top decile (the 10th decile in Figure 5), the LSS model outperformed the FIB-4 and NFS models, as well as the waist circumference classification method, by over 42.6%, 37.7%, and 29.5% in screening for F3 + F4 and by 38.9%, 44.4%, and 47.2% in screening for F4, respectively. Additionally, the waist circumference classification method showed better performance than the FIB-4 and NFS models for screening F3 + F4 and F2–F4 fibrosis.

### 5.3. Possible Clinical Applications of LSS Model

The approach of calculating the risk probability of fibrosis in patients in the LSS model, instead of a direct cutoff value, seems promising. It offers a flexible approach with relatively good accuracy, calculating probabilities below 75% for diagnosing F2–F4, 50% for diagnosing F3 + F4, and 30% for diagnosing F4, as observed in Figure 2 and tested in Figure 3. To address the overestimation of risk beyond these ranges, adjustments can be made based on the patient’s risk probability. For example, if the risk probability of F3 + F4 falls between 50 and 80%, a correction of 10% can be subtracted from the estimation. Similarly, for screening the risk of F4, a correction of 10–15% can be subtracted for risk ranges above 45%, as shown in Figure 3.

Utilizing variables collected during routine office visits and clinical laboratory work, the LSS formula and probability formulas may offer practitioners a useful alternative method for screening fibrosis in at-risk patients. The new LSS model favors body weight and AST over waist circumference and GGT because these variables are more commonly measured in routine clinical settings. If the calculated risk of fibrosis for a patient is deemed unacceptable, referral to a liver specialist can be made for further evaluation, including the use of FibroScan and appropriate care. Additionally, for patients with initially low LSS values, follow-up tests showing an increasing LSS and thus an increasing risk of fibrosis may warrant reconsideration for referral. Establishing minimum LSS values and risk levels of fibrosis for referral guidelines, agreed upon by an expert group, could further assist practitioners in their decision-making process.

## 6. Limitation

Limitations of this study include the fact that the LSS model is designed for screening at-risk patients rather than providing a definitive diagnosis, necessitating follow-up with methods such as FibroScan or liver biopsy if needed, as per AGA/AASLD guidance. Additionally, regression models typically only capture trends, not variations, which may limit the utility of our new model. This study is also limited by the reliance on FibroScan results; despite its good reliability, it is still considered inferior to liver biopsy, which remains the gold standard for diagnosing fibrosis. Despite these limitations, this study boasts several strengths. It was validated using FibroScan LSM data from NHANES 2017–2018, which include a large, ethnically diverse sample of US adults, enhancing the generalizability of the results. Additionally, NHANES data quality is generally well regarded, suggesting robust findings.

## 7. Conclusions

The new LSS screening model, along with its probability formula, may offer a useful alternative method for screening fibrosis in the population at risk of MASLD. Derived from the relationship between LSM, body measurements, and biochemistry data of 2414 at-risk NHANES participants, it was validated against a separate dataset of 1562 at-risk NHANES participants identified following AGA/AASLD guidance. The LSS model had AUROCs of 0.79 and 0.78 for the diagnosis of cirrhosis (F4) and advanced fibrosis (F3 + F4), respectively. In comparison, the NFS model exhibited AUROCs of 0.76 and 0.7, while the FIB-4 model had AUROCs of 0.71 and 0.61 for the diagnosis of F4 and F3 + F4, respectively. The top decile of the LSS model improved the screening accuracy for potential fibrosis by 38.9%, 44.4%, and 47.2% for F4 and 42.6%, 37.7%, and 29.5% for F3 + F4, compared to the top deciles of the FIB-4 and NFS models, as well as waist circumference classification alone. The LSS model also outperformed the two recent models, SAFE and FIB-3, in screening for fibrosis in the at-risk population. This suggests that the LSS model and its probability formula, utilizing only routine laboratory and clinical data, could offer an alternative and potentially useful method for fibrosis screening in the at-risk adult population.

## Figures and Tables

**Table 1:** Characteristics of NHANES participants aged 18 to 80 years old for the 2017–2018 and 2017–2020 sample cycles.

Average Age, Body Measurements, and Blood Chemistry Data Examined					
	2017–2020	2017–2018		2017–2020	2017–2018
Age (years)	55.8 ± 0.3	56.1 ± 0.4	GGT (IU/L)	38.5 ± 1	38.7 ± 1.2
Waist circ. (cm)	90.8 ± 0.5	89.7 ± 0.6	Platelet count (1000 cells/UL)	244.3 ± 1.4	239.7 ± 1.7
Weight (kg)	108.3 ± 0.3	107.8 ± 0.4	Albumin (g/dL)	40.4 ± 0.1	40.3 ± 0.1
BMI			Triglycerides (mg/dL)	171.9 ± 2.4	180.5 ± 3.3
HbA1C (%)	6.4 ± 0.03	6.4 ± 0.03	HDL		
ALT (IU/L)	27.8 ± 0.4	28.1 ± 0.5	Systolic BP (mm Hg)	131.8 ± 0.4	132.6 ± 0.5
AST (IU/L)	23.8 ± 0.3	24.3 ± 0.4	Diastolic BP (mm Hg)	75.6 ± 0.2	74.2 ± 0.3
Counts and percentages of fibrosis stage			Counts of races		
^1^ Fibrosis score	2017–2020	2017–2018		2017–2020	2017–2018
F0-F1 (LSM < 7.1)	1870 (77.5%)	1205 (77.1%)	Mexican Americans	331	244
Ft (7.1–7.5)	72 (3%)	43 (2.8%)	^2^ NMA Hispanic	281	163
F2 (7.5–10)	247 (10.2%)	167 (10.7%)	White	767	497
F3 (10–14)	126 (5.2%)	81 (5.2%)	Black	615	342
F4 (>14)	99 (4.1%)	66 (4.2%)	Asian	308	233
Total	2414	1562	Others	112	83
Counts of men in two sample cycles			Counts of women in two sample cycles		
men	1320	841	women	1094	721

Note: The table shows the mean ± standard error for continuous variables and counts per group for categorical variables. Waist circ: waist circumference. ^1^ Fibrosis Score classified based on FibroScan LSM values: F0–F1: 2–7 kPa; Ft: 7–7.5 kPa; F2: 7.5–10 kPa; F3: 10.1–14 kPa; F4: >14 kPa. ^2^ NMA: Non-Mexican American Hispanic.

**Table 2:** AUROC values for F4, F3 + F4, and F2–F4 diagnoses of 2017–2018 NHANES at-risk participants: comparison of the new LSS and existing models.

	F4	F3 + F4	F2–F4
LSS	0.79 (0.73–0.86)	0.78 (0.74–0.82)	0.73 (0.7–0.77)
NFS	0.76 (0.7–0.81)	0.7 (0.65–0.74)	0.65 (0.62–0.69)
FIB-4	0.71 (0.64–0.78)	0.61 (0.56–0.67)	0.57 (0.53–0.61)
SAFE	0.77 (0.7–0.84)	0.71 (0.66–0.76)	0.67 (0.63–0.7)
FIB-3	0.75 (0.68–0.81)	0.65 (0.6–0.7)	0.59 (0.55–0.63)

## Data Availability

The data used in analyses in this study are available at https://wwwn.cdc.gov/nchs/nhanes/search/datapage.aspx?Component.

## Abbreviations

AASLD	American Association for the Study of Liver Diseases
AGA	American Gastroenterological Association
ALT	Alanine transaminase
AST	Aspartate transaminase
AUROC	Area under the receiver operating characteristic curves FIB-4: Fibrosis-4
FIB-3	Fibrosis-3 Index
FIB-4	Fibrosis-4 (FIB-4)
HDL	High-density lipoprotein
IQR	Interquartile range
LSM	Liver stiffness measurements
LSS	A liver stiffness score
MASLD	Metabolic dysfunction-associated steatotic liver disease
NAFLD	Non-Alcoholic Fatty Liver Disease
NFS	NAFLD Fibrosis Score
NHANES	National Health and Nutrition Examination Surveys
SAFE	Steatosis-associated fibrosis estimator
